# Predicting live birth chances for women with multiple consecutive failing IVF cycles: a simple and accurate prediction for routine medical practice

**DOI:** 10.1186/1477-7827-11-1

**Published:** 2013-01-09

**Authors:** Géraldine Porcu, Philippe Lehert, Carolina Colella, Claude Giorgetti

**Affiliations:** 1 Institut de Médecine de la Reproduction, Marseille, F-13417, France; 2 Faculty of Economics, University of Louvain, Mons, B-7000, Belgium; 3 Faculty of Medicine, University of Melbourne, Melbourne, 3010, Australia; 4 Medical Advisor Fertilility, Merck Serono s.a.s., 37 rue Saint-Romain, Lyon cedex 08, F-69379, France

**Keywords:** IVF, ICSI, Predictive model

## Abstract

**Background:**

Women having experienced several consecutive failing IVF cycles constitute a critical and particular subset of patients, for which growing perception of irremediable failure, increasing costs and IVF treatment related risks necessitate appropriate decision making when starting or not a new cycle. Predicting chances of LB might constitute a useful tool for discussion between the patient and the clinician. Our essential objective was to dispose of a simple and accurate prediction model for use in routine medical practice. The currently available predictive models applicable to general populations cannot be considered as accurate enough for this purpose.

**Methods:**

Patients with at least four consecutive Failing cycles (CFCs) were selected. We constructed a predictive model of LB occurrence during the last cycle, by using a stepwise logistic regression, using all the baseline patient characteristics and intermediate stage variables during the four first cycles.

**Results:**

On as set of 151 patients, we identified five determinant predictors: the number of previous cycles with at least one gestational sac (NGS), the mean number of good-quality embryos, age, male infertility (MI) aetiology and basal FSH. Our model was characterized by a much higher discrimination as the existing models (C-statistics=0.76), and an excellent calibration.

**Conclusions:**

Couples having experienced multiple IVF failures need precise and appropriate information to decide to resume or interrupt their fertility project. Our essential objective was to dispose of a simple and accurate prediction model to allow a routine practice use. Our model is adapted to this purpose: It is very simple, combines five easily collected variables in a short calculation; it is more accurate than existing models, with a fair discrimination and a well calibrated prediction.

## Background

Methods to predict chances of live birth (LB) have recently attracted attention in assisted reproductive technology (ART) research. As ART techniques have potential side effects and are costly, before deciding on IVF (*in vitro* fertilisation), it remains essential to estimate the chances of success. In particular, it has been suggested that ART should only be used for couples with a prognosis that significantly exceeds the expected success rate in the absence of treatment [1].

The complex multi-factorial genesis of infertility prevents clinicians from offering an accurate, reliable prognosis [2]. Hence, researchers have developed predictive models in which the LB rate is predicted from the mix variables (couple baseline demographic and clinical characteristics and historical fertility data). However, these models cannot be considered as an infallible panacea: Model-based predictions can be biased, inaccurate or non-generalizable [3]. A careful review of these models [4] revealed that only a few can be considered as a guiding tool in making decisions about fertility treatment in patient couples similar to the development population. However, these models lack discrimination and calibration as to be used in routine medical practice. Templeton’s model [5] for IVF, Hunault’s model [6] for treatment-independent pregnancy and Steures’ model [7] for IUI (intra-uterine insemination) were considered as adequately validated. Templeton model is the most widely used, despite a lack of accuracy, and poor performances in external validation [8,9].

A first reason of the poor performances of models is the considerable influence of the ART centre, expected to be even more determinant than the patient mix [3]. The centre effect combines several interrelated factors related to centre-selected treatments and procedures and variable skill levels. A second reason is that the models were limited on historical data for which the available variables were defined *a priori* by health authorities. A third reason is the rapid evolution of techniques making a model obsolete some years after its development. To alleviate these problems, Arvis *et al*. [10] have suggested modification of Templeton according to a centre specific fitting approach accounting for trend effect, and suggest to add new predictors (BMI, FSH and Smoking habits) in the model. In using these changes, a much better discrimination and calibration was observed, and found appropriate for routine practice use.

A last reason avoiding precision of the currently proposed models is their generality, i.e. their ability to be used for any centre and any type of patients. Among the numerous possible sub-groups to consider, the set of women having experienced several consecutive failed cycles (CFCs, i.e. not resulting into a live birth) are of particular interest. We concentrated on this subset for three main reasons: a) For these patients, the decision to start a new IVF cycle is particularly crucial, given the previous failures, and the increasing perception of doubtful Risk/Benefit ratio. b) In some countries, the health insurance system compensates costs for a fixed maximum number of cycles, which severely affects the patient Cost/benefit ratio, when this number is exceeded. c) Compared with patients entering a first cycle, these patients are documented by many more potential available predictors, in particular all the intermediate and end-stage measurements of the previous failing cycles (such as number of oocytes, embryos and their quality). Thus using these measurements closer of the studied endpoint should logically provide a much higher precision of the prediction.

In France, the Health insurance reimburses four cycles at the most. The occurrence of women with four CFCs is not rare in our centre, and the concern and uncertainty of these patients to embark or not a new cycle appears such a crucial issue that we decided to perform a specific research on this subgroup.

## Methods

Our site performs about 1600 IVF/ ICSI (intracytoplasmic sperm injection) procedures and 1000 IUI cycles per year. Since 2000, we record the characteristics of each cycle in terms of the patient mix, treatments, biological and clinical treatment outcomes. As the study was purely observational and non-interventional, it was not necessary to declare it to any Ethics Committee. Clinical characteristics for all these patients were as follows: Pituitary desensitization was treated with GnRH (gonadotropin-releasing hormone) agonist or antagonist protocols with daily subcutaneous administration of either 0.25 mg of cetrorelix (Cetrotide®, Merck Serono, Geneva, Switzerland), 0.25 mg of ganirelix (Orgalutran®, Schering-Plough, Kenilworth NJ, USA) or 3 mg of long-acting cetrorelix (Cetrotide®, Merck Serono). The starting doses of recombinant FSH (follicle-stimulating hormone) and either follitropin beta, highly purified gonadotropin, or human menopausal gonadotropin ranged from 112.5 to 225 IU/day for patients with normal ovarian reserve. hCG was administered once three follicles reached a mean diameter ≥ 17 mm. Oocyte retrieval was performed 35–36 hours after hCG injection by transvaginal, ultrasound-guided follicular aspiration. For conventional IVF or ICSI, fertilization was confirmed 18 hours after insemination. Embryos were classified according to their morphological appearance using an embryo scoring system (0 to 4 points) [11]. The luteal phase was supported by intravaginal administration of 400 mg of micronized progesterone per day. Clinical pregnancy was confirmed when ultrasonography performed approximately 5 weeks after transfer demonstrated the presence of at least one gestational sac.

### Statistical analyses

We conducted a mixed linear model to assess the variation of the number of oocytes, embryos, good quality embryos, and gestational sacs during the four CFCs. Our primary endpoint was the occurrence of at least one LB. Our study population consisted of patients with at least four CFCs, and undertaking a new cycle, all of then performed in our centre, without any other selection. A logistic model was built with a stepwise strategy based on minimization of the Akaike information criterion (AIC [12]), considered as an appropriate trade-off between determination and parsimony. The stepwise approach was carried out during an expert meeting in which the choice of predictors was discussed from both clinical (literature search) and statistical (p-values and AIC) standpoint. Given the expected small sample size, exact tests were scheduled to assess the significance [13]. The candidate predictors were specified *a priori* and all the variables mentioned in previous models [4] were available in our database.

The model calibration (goodness of fit between observed and expected (predicted) values of the observed response, irrespective of prediction level) was assessed by Hosmer and Lemeshow goodness of fit test [14]. Goodness of fit was determined by the C-statistic (the area under the ROC curve). Prognostic parameters are presented as the odds ratio (OR) [95% confidence interval (CI)] relative to the indicated reference category. For sensitivity purposes and facilitating clinical interpretation, we compared the logistic model, with an ordinary least squares OLS linear model adapted for binary endpoints, where effects can be interpreted as absolute risk difference per unit.

All statistical analyses were carried out with R software (release 2.12.1).

## Results

Over the period 2001–2006, we identified 151 women with at least five consecutive IVF cycles of which the first four failed. Fifty-two (34%) of the 151 women were under 35 years of age. Women under and over 35 years were divided into subgroups and compared in terms of patient mix variables and ART values (Table 1). The main reasons for couple infertility were male infertility (43%) and tubal abnormalities (30.5%). The mean number of oocytes, embryos and good-quality embryos in the first four cycles were all higher in women below 35 years than in those aged 35 and over. For the 5^th^ cycle, the LB pro-portions were 36.5% and 22% in the two groups, respectively, and 27% (range: 20%; 34%) for the whole sample.

**Table 1:** **Sample distribution and comparisons between women** <**35 and** ≥**35 years of age**

**Cause of infertility**	<**35 years**(**N**=**52**)		≥ **35 years**(**N**=**99**)		**Total **(**N**=**151**)	
· *Male infertility*	30	57.7%	35	35.4%	65	43.0%
· *Endometriosis*	1	1.9%	6	6.1%	7	4.6%
· *Unexplained*	5	9.6%	14	14.1%	19	1.6%
· *Tubal abnormalities*	14	26.9%	32	32.3%	46	30.5%
· *Viral risk*	2	3.8%	12	12.1%	14	9.3%
*Mean baseline FSH (IU/L)*	6.50	± 2.79	7.11	± 2.57	6.90	± 2.65
*Mean ± SD number of oocytes*	7.91	± 3.40	6.79	± 2.96	7.17	± 3.15
*Mean ± SD total number of embryos*	5.24	± 2.57	4.76	± 2.13	4.92	± 2.30
*Mean ± SD number of good-quality embryos*	1.08	± 0.77	0.84	± 0.60	0.92	± 0.67
*Women with at least one gestational sac (%)*	37.5%		23.3%		28.2%	
*Women with at least one foetal heart beat (%)*	37.0%		22.9%		27.8%	
*LB rate*	36.5%		22.2%		27.2%	

Values are given as numbers and percentages (%) or the mean ± SD. FSH: follicle-stimulating hormone (iu/L). LB: live birth.

### Main predictive model

For each patient, the mean number of oocytes, embryos, good-quality embryos remained constant over the 4 studied cycles (Mixed ANOVA, p<0.35 for all series).

Our stepwise strategy identified five significant predictors (Table 2). The most determinant predictor was the occurrence of at least one Gestational Sacs OGS observed over the 4 last observed cycles, with an odds ratio (OR) of 3.09 (95% CI [1.39; 6.88]; p=0.005). The next identified predictor was the mean number of good-quality embryos over the four cycles (MQE) with another very positive effect on LB (OR=2.42; 95% CI [1.32; 4.43]; p=0.004). Age was found as third predictor (age-25 was used to define the reference population) with a linearly decreasing effect on LB (OR=0.87; 95% CI [0.78; 0.96]; p=0.008). Male infertility aetiology was found with a positive effect on LB (OR=3.86; 95% CI [1.09; 13.5]; p=0.005). Finally the binary variable FSH10 (defined as 1 or 0 following that FSH was less or exceeded 10 IU/L) was the last significant predictor with a positive effect on LB (OR=4.22; 95% CI [1.26; 14.7]; p=0.019). The intercept value of the model was 0.044 (95% CI [0.006; 0.392]; p=0.004), provides an estimate of 4.4% of success rates for a standard population defined such that all the predictors are fixed to 0, thus women aged of 25 years, with FSH>10, with any aetiology except male infertility, without any cycle with at least one gestational sac and no good quality embryo.

**Table 2:** The main predictive data from the logistic regression model

**Effects**	**Logistic Model**				**Linear Simplified Model**			
	**OR**	**95% CI**		**P**	**Incr**	**95% CI**		**P**
*Intercept*	0.044	0.006;	0.392	0.004	0.045	0;	0.350	0.047
*Women age*	0.868	0.782;	0.964	0.008	−0.023	−0.039;	−0.006	0.009
*FSH < 10 IU/L*	4.222	1.267;	14.077	0.019	0.200	0.039;	0.361	0.016
*Male Infertility Etiology (MI)*	3.862	1.097;	13.595	0.035	0.172	0.011;	0.334	0.038
*occurrence of at least one GS (OGS)*	3.099	1.395;	6.884	0.005	0.174	0.047;	0.302	0.008
*Mean number of GQE (MQE)*	2.422	1.322;	4.437	0.004	0.168	0.064;	0.263	0.003

Proportion of variability due to covariates R^2^=0.2955; ROC curve AUC (C) = 0.76 (95% CI [0.71–0.81]) and the simplified model (from an ordinary least squares procedure suited to binary data). OR: odds ratio; CI: confidence interval. Logistic model coefficients can be assimilated to OR, and linear model coefficients to incremental values (Incr). For interpreting OR and Incr, age, number of cycles with at least one sac, and mean number are continuous values, while FSH<10 IU/L and Male infertility are binary values.

All the other variables were found as not determinant: the number of embryos transferred, secondary vs. primary infertility, GnRH down-regulation agonist or antagonist, FSH supplementation, stimulation duration (mean value over all cycles and the separate values for each cycle), hCG supplementation, hCG dosage, mean total number of oocytes and mean total number of embryos.

The model discrimination was studied with a ROC curve (C-statistic =75.4; 95% CI [70.1; 80.8]). The calibration was examined by calculating the difference between the observed and predicted probabilities within 6 categories of increasingly high estimated probabilities. Hosmer-Lemeshow’s test did not reject the hypothesis of goodness of fit between the observed and predicted values (p=0.651).

For sensitivity purpose, we conducted an alternative OLS (ordinary least square) model which derived very similar results (Table 2). Compared with the logistic model, this expression was characterized by a loss of discrimination (C=72.2; 95% CI [66.1, 77.3]).

## Discussion

Predicting Live Birth chances for a couple consulting for sub-fertility is nowadays considered as an important step before decision to begin an IVF cycle. But for some subgroups of patients, the a priori knowledge of LB probability seems more important to know: This is particularly the case when a couple has experienced several failed cycles, resulting to a growing perceived concern of irremediable failure, increasing in the same time IVF related side effects and costs. The final decision to start a new cycle must be discussed between the clinician and the couple. In this context, a prediction model based on historical experience of this couple constitutes an appropriate quantitative and objective measurement tool. This model must obviously be accurate, thus we tried to optimize its precision in restraining our population to patients with multiple failures, specific to our centre in particular.

### Clinical findings and comparison with previous models

Our results may usefully be compared with those from other models that are applicable to the general population. Age remains a determinant factor and was associated with a linear decrease in fertility. Due to the expected low proportion of young (<30 years) women in this category, the non-linear effect of age found in other models [5,10] was not confirmed. Male infertility was already identified as the infertility cause associated with better outcomes opposed to tubal problems, which has a particularly negative prognosis [9,15]. High basal FSH values were recently considered as a prognostic factor for a poor ovarian response [10,16], and the same cut-off critical value of 10 IU/L was confirmed.

However, the most important predictors were OGS (one gestational sac) and MQE. MQE was already suggested in previous researches [17], it constitutes an intrinsic, woman-specific quality characteristic of oocyte production, confirmed by our analysis demonstrating a constant value of the number of quality embryos across cycles. OGS may additionally be related to independent paternal effects and implantation quality [18] for which other measurements (endometrial thickness, in particular) were not available. OGS takes into account the oocyte quality, the quality of the partner’s genetic information and the endometrial receptivity [19]. Lastly, given that the gestational sac is and end-stage in the ART process, its high determination on LB was expected.

### Model performance

Fixing all the predictors to zero in our model (Table 2) characterizes a population of 25 years old, FSH>10 IU/L, any aetiology except male infertility, and no good quality embryo or gestational sac obtained during the last cycles. Except for age, this reference population corresponds to the worst case or most unfavourable case in terms of LB success. The intercept value of our model provides a very low estimate of LB of 4.4%, and this value is even expected to decrease with increasing age. However, it may considerably increase depending on the values of the four predictors (OGS, MQE, FSH10 and MI). As a corollary, if at least one of these conditions is not fulfilled, the predicted LB rate is close of 0%, thus the interest of conducting IVF is very limited, at any age.

Our linear model (Table 2) allows to apprehend the high variation of prediction: Compared with the reference population, a value of FSH<10 contributes to an LB increase of 20%, whereas, each of the conditions MI, OGS and MQE increments LB probability of around 17%, thus a couple characterized by the four conditions may be predicted with a probability as high as 20+3*17=74%. Whereas the reference population is characterized by a virtually null probability of live birth, depending on the four predictors may dramatically make vary LB. In particular, couples characterized by at least one of the positive predictors (MI, OGS, MQE, FSH10) correspond to LB predictions of at least 17%, a value not far from observed LB rates in normal populations.

These results highlight the importance of individual prediction: Based on this sample, we found an LB rate of 27% (range: 20%; 34%), which seems to show that even after four CFCs, the LB probability remains non-negligible. However, this value is extremely varying among patients, depending on a small number of variables included in our model.

### Model sensitivity and accuracy

**Figure 1:**
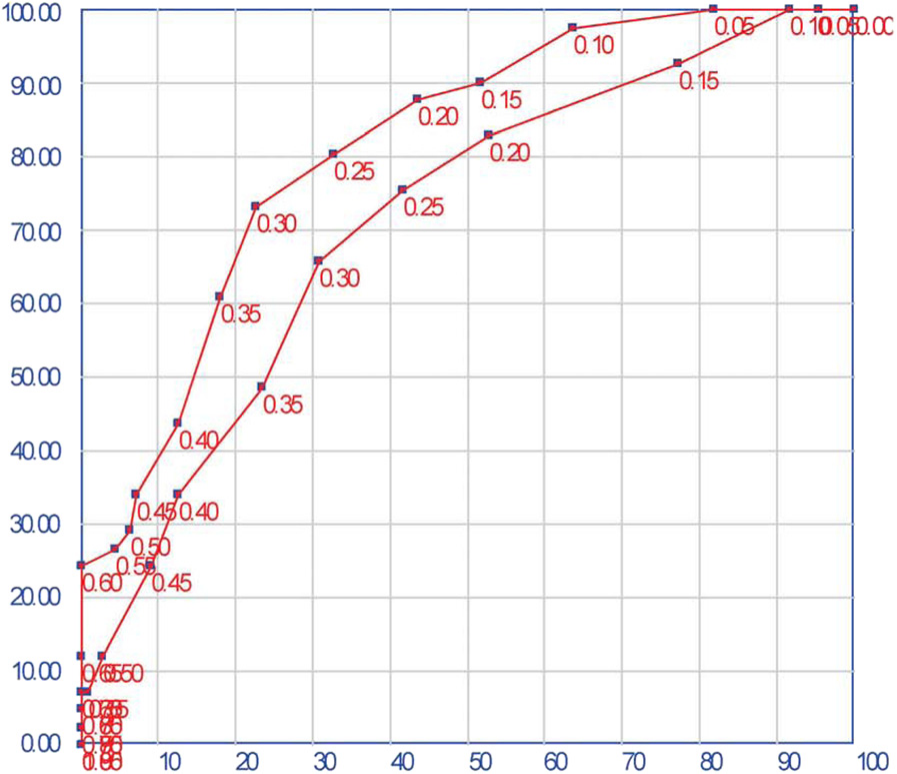
**The ROC curves associated with the primary model.** (C-statistic: 0.76, [0.71–0.81]) and the simplified model (C- statistic: 0.66, [0.61–0.72]).

A model is considered to have poor, fair or good performance levels if the C statistic lies between 0.5 and 0.7, 0.7 and 0.8 or 0.8 and 0.9, respectively [20]. Our C-statistic of 0.76 (95% CI [0.71; 0.81]) shows that performance was fair and markedly better that the poor discrimination exhibited by Templeton model, (0.56 < C < 0.63) (See Figure 1). A recent attempt to model LB in a single centre by using embryo quality as the main predictor [21] resulted in similarly high discrimination (C=0.77). We attribute this improvement to the strong predictive value of OGS and MQE, parameters which are biologically and chronologically closer to the endpoint (LB) than the baseline parameters used in Templeton model.

### External validation, generalizability and implication for practice

Our objective was to develop a prediction model for patients characterized by multiple CFCs. As we initially conjectured it, this model was found more determinant than models set up on general population, and thus it is appropriate for use in routine medical practice. This model is very simple, only requires 5 values easy to retrieve, and a simplified calculation can be easily derived from our linear model.

This model was developed in one centre, the question as to which extent can such results be useful for other centre needs in principle an external validation. Our results might probably constitute a good approximation.

Given the high between-centre variability observed in the literature, a centre-specific model is probably the simplest, most powerful and highly feasible solution [10]. Ideally, the model can be re-fitted to the specific data of a centre in particular. Fitting a logistic regression is an easy task, including complementary techniques to preserve stability and reduce the overfit of the model. However, the number of patients with multiple CFCs is not so common, and a sufficient sample size will rarely be reached except for large centres. Moreover, inter-centre coordination should be necessary for the very common case in which patients are switching to other centres after some negative experience.

In spite of this possibility, we argue that our model can be considered as a good approximation: The invariance of the Templeton model was recently demonstrated from adapted external validations [10]: The coefficients mix-related remained comparable among centres, across various cultures, countries and types of hospitals, only the intercept value was found much different across centres, in estimating the centre performance (mean LB rate). Our model uses essentially the same variables thus we argue that the coefficients of these variables must remain stable across centres. The intercept of the model was very small and corresponds to LB rate in the most unfavourable populations, this value should remain very low, irrespective of centres. In total, as the intercept is negligible, our model should constitute a fair approximation, when applied to other centres. Finally, our linear model, although slightly less accurate allows a very easy calculation of LB: Starting from a virtually null probability of LB, adding 20% when FSH<10, adding 17% for each of the three positive conditions (MQE, OGS, NI) and subtracting 2% for each year in excess of 30. As an example, LB probability for a 40 years women of observed with FSH<10 and a mean number of QE for every cycle should be 20+17-2*(40–30)=17%.

### Study limitations

Our dataset was not a random dataset and not all the patients with at least four CFCs were treated, a part of these couples dropped out and renounced to their fertility project. It is probable that more favourable prognosis were treated, thus our estimate compared with the strict population should be over-estimated on a strict Intent to Treat basis. In that sense, our research has not the objective to determine an estimate of LB rate in the population of couples with multiple CFCs. Malizia *et al*. [22] clearly underlined the difference between patient returning to IVF, and those interrupting their project, in showing the poorer prognosis of the latter group. As a consequence, for optimistic and conservative estimates, we calculated in censoring non-returning patients or assimilating them as failures, respectively. Our objective was to predict LB chances for patients returning in our centre to evoke a new possible IVF cycle, thus a correction for non-returning patients is not necessary. However, the proportion of LB found in our study cannot be assimilated to the whole population, but only to returning patients.

Another study limitation related to our relatively small sample size. However, the number of patients with 4 CFCs is not so common, and requires a large recruitment to remain in one centre. Moreover, when facing failures, many couples switch to other centres, which makes their follow-up very difficult to maintain. We intend to improve our model in extending results with a collaborative multicentre regional study, in federating neighbour sites to easily dealing with between-centres patients’ switches. We also plan to use an adaptive technique to account for new patients and add variables that were not documented when we started the project. On the basis of our current C-statistic of 0.76, first new calculations should allow an increase of model discrimination until C=0.8, thus provide an even more precise prediction.

## Conclusions

Women with multiple failing cycles constitute a critical and particular subset, for which growing perception of irremediable failure, increasing costs and drug-risks necessitate a precise measurement tool of success, before starting a new cycle. Our essential objective was to dispose in routine medical practice of a simple and accurate prediction model, so far not available from previous researches. In taking advantage of more discriminatory variables such as the number of gestational sacs or good quality embryos, we provide an easy rule enabling a simple calculation of LB probability, combining five simple individual characteristics, presumably considered as a good approximation to any centre in particular.

## Competing interests

PL, GP, CG have no conflicts of interest to declare with respect to the present work. CC currently works for Merck Serono.

## Authors’ contributions

GP and CG contributed to study conception and design, interpretation and critical revision of the article. PL conducted the statistical analysis interpretation and drafting and critical revision of the article. CC was involved in the review of the literature and critical revisions of the article. All authors read and approved the final manuscript.
